# Supplementary material for: The genome sequence of the Royal Burnet, Zygaena sarpedon Hübner, 1790 (Lepidoptera: Zygaenidae)
Source: Wellcome Open Res. 2026 Sep 11;11:635. [Version 1] doi: 10.12688/wellcomeopenres.27779.1 (PMC13617277; doi:10.12688/wellcomeopenres.27779.1)
Supplement: Peer review history

# Reviewer report: Reviewer response for version 1

**DOI:** 10.21956/wellcomeopenres.30570.r168452

**Published:** 2026-09-28

**Authors:** Gen Morinaga

**Affiliations:** 1 University of Calgary, Calgary, Alberta, Canada

In the genome note titled “The genome sequence of the Royal Burnet, *Zygaena Sarpedon* Hubner, 1790 (Lepidoptera: Zygaenidae)”, Vila et al., combine PacBio HiFi sequencing with Hi-C sequencing to output a highly complete, highly contiguous chromosome scale assembly (haplotype 1), and a slightly less contiguous scaffold-level assembly (haplotype 2). They identified the Z sex chromosome and assembled the mitogenome as well (included as part of haplotype 1). They also include a map of the Merian elements identified in the chromosomes in haplotype 1.

 The work presented is done to a high standard and uses state-of-the-art sequencing techniques and bioinformatics. The methods are adequately presented and documented. The writing is generally clear, but the authors use the passive voice over an active one (e.g., “The HiFi reads were assembled…” as opposed to “We assembled the HiFi reads…”). While not necessary for this manuscript, in future works, I would suggest to the authors to switch to an active voice because it helps to make the manuscript easier to read/follow and often helps to shorten sentences.

Are the datasets clearly presented in a usable and accessible format, and the assembly and annotation available in an appropriate subject-specific repository?

Yes

Are sufficient details of the sequencing and extraction, software used, and materials provided to allow replication by others?

Yes

Are the rationale for sequencing the genome and the species significance clearly described?

Yes

Are the protocols appropriate and is the work technically sound?

Yes

Reviewer Expertise:

Comparative genomics, genomics

I confirm that I have read this submission and believe that I have an appropriate level of expertise to confirm that it is of an acceptable scientific standard.

# Reviewer report: Reviewer response for version 1

**Published:** 2026-09-11

**Authors:** Automated Benchmarking

**Affiliations:** 1 Wellcome Sanger Institute, Hinxton, UK

As this article is a Genome Note, Automated Benchmarking has been used to determine whether the reported assembly passes three defined checks established by the Earth BioGenome Project: the raw and assembled data are publicly available, the Earth BioGenome Project three-part metric is met, and the BUSCO score passes the stated threshold. For Zygaena sarpedon (ilZygSarp1), all three checks passed. Data available: pass. ENA study PRJEB81941, raw-read accessions ERR13917241 and ERR13925963, and assembly accessions GCA_964338965.1 and GCA_964339015.1 are public. Earth BioGenome Project three-part metric: pass. Haplotype 1 is reported as 6.C.Q66, meeting the 6.C.Q40 reference standard. BUSCO: pass. Haplotype 1, which is proposed as the reference assembly for this species, has 97.9% complete BUSCOs (97.5% single-copy and 0.4% duplicated). It meets the target of greater than 90% complete and single-copy BUSCOs. This decision uses values reported in the Genome Note and public accession records.

Reviewer Expertise:

NA

I confirm that I have read this submission and believe that I have an appropriate level of expertise to confirm that it is of an acceptable scientific standard.
